# Resilin-mimetics as a smart biomaterial platform for biomedical applications

**DOI:** 10.1038/s41467-020-20375-x

**Published:** 2021-01-08

**Authors:** Rajkamal Balu, Naba K. Dutta, Ankit K. Dutta, Namita Roy Choudhury

**Affiliations:** 1 Chemical and Environmental Engineering, School of Engineering, RMIT University, Melbourne, VIC 3000 Australia; 2 Department of Medical Oncology, Dana-Farber Cancer Institute, Harvard Medical School, Boston, MA 02115 USA

**Keywords:** Drug delivery, Biomaterials, Bioinspired materials, Biomedical materials

## Abstract

Intrinsically disordered proteins have dramatically changed the structure–function paradigm of proteins in the 21^st^ century. Resilin is a native elastic insect protein, which features intrinsically disordered structure, unusual multi-stimuli responsiveness and outstanding resilience. Advances in computational techniques, polypeptide synthesis methods and modular protein engineering routines have led to the development of novel resilin-like polypeptides (RLPs) including modular RLPs, expanding their applications in tissue engineering, drug delivery, bioimaging, biosensors, catalysis and bioelectronics. However, how the responsive behaviour of RLPs is encoded in the amino acid sequence level remains elusive. This review summarises the milestones of RLPs, and discusses the development of modular RLP-based biomaterials, their current applications, challenges and future perspectives. A perspective of future research is that sequence and responsiveness profiling of RLPs can provide a new platform for the design and development of new modular RLP-based biomaterials with programmable structure, properties and functions.

## Abstract (web summary)

Advances made in synthesis and analytical techniques has allowed the exploration and mimicry of natural materials. Resilin-mimetics have emerged from this advance as a biomaterial with a range of potential applications. Here, the authors review the history and current research on resilin-mimetics, providing a future perspective.

## Introduction

Native elastomeric proteins are biomaterials that have been perfected over billions of years by natural selection to act as molecular springs in a wide range of biological systems to drive unique functions. Among native proteins, resilin is purported to be one of the most efficient elastic proteins known. It is essentially a structural protein, which exists mainly in insect exoskeleton structures and exhibits outstanding resilience and fatigue life^1^. The first description of resilin was made in 1960s as a rubber-like protein observed in locust-wing hinge and dragonfly tendon^2^. Early studies on the composition and structure of resilin revealed the protein to contain about 66% hydrophobic residues (much lower than elastin) with about 45% proline and glycine residues combined^3^. In native state, resilins exist as di- and trityrosine crosslinked hydrogels, and exhibit highly amorphous structure when examined using X-ray diffraction and electron microscopy^4,5^. During biosynthesis, pro-resilins (uncrosslinked) are secreted from the apical surface of the epidermal cells into the subcuticular space, where they are crosslinked by an enzyme-mediated process to form hydrogels^6^. Over the course of next three decades, resilin was also identified in many other insects and arthropods, including copepods^7^, reduviidae^8^ and moth^9^. In arthropods, resilin is largely involved in a number of different functions, including the flexibility and deformability of membranous cuticle and joint systems, the storage of elastic energy in locomotion (jumping, flying, etc.) and catapulting systems, the adaptability to surface topography by multiple contact attachment, and prey catching systems and the reduction of fatigue and damage in feeding and traumatic reproductive system^10^.

**Fig. 1:**
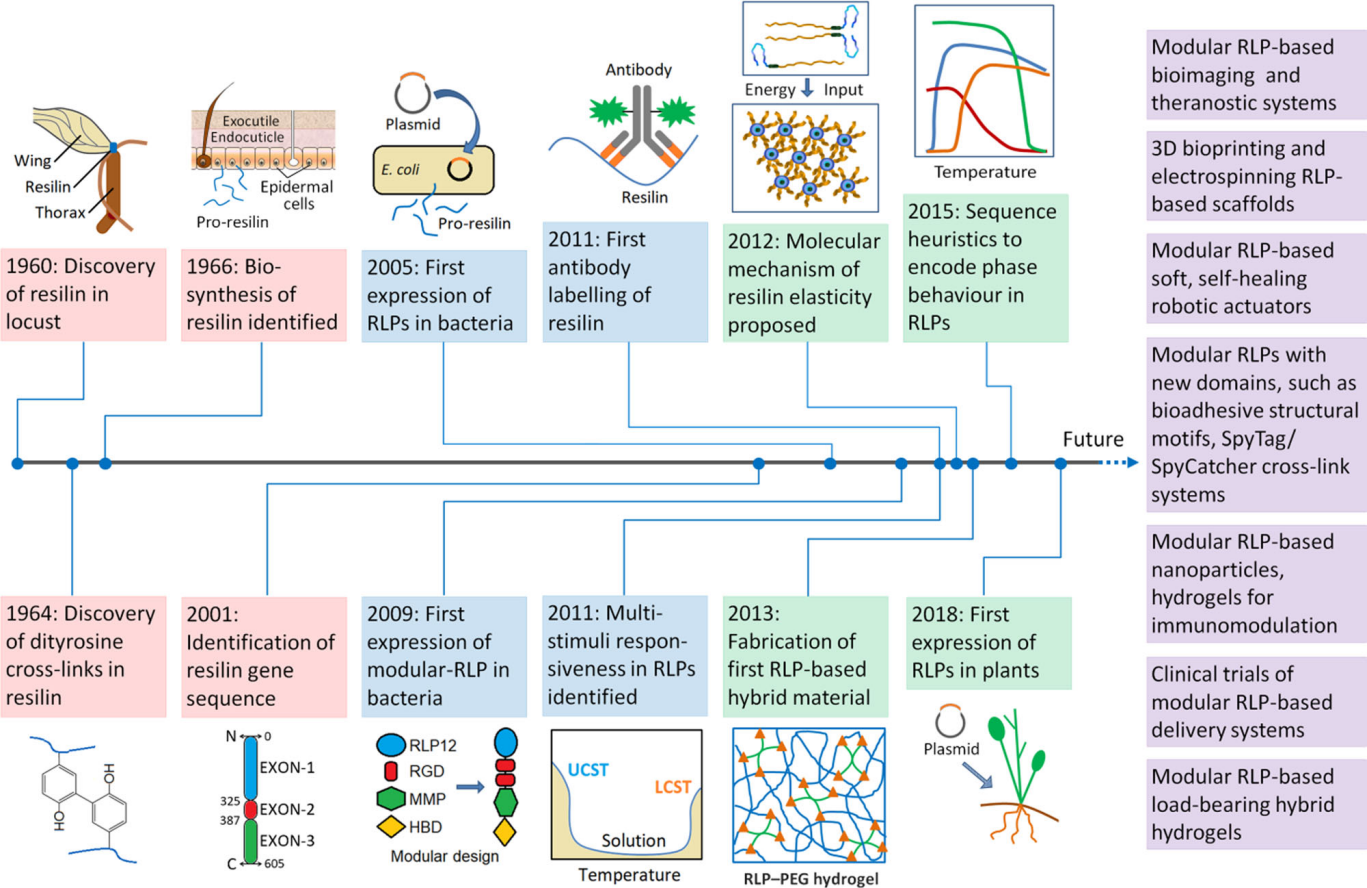
Timeline of key discoveries and milestones of resilin and RLP-based biomaterials. Description of illustrations (in the order of year): 1960—locust-wing hinge showing resilin, 1964—covalent dityrosine crosslinks in native resilin, 1966—native uncrosslinked pro-resilin synthesized by epidermal cells into the subcuticular space, 2001—full-length resilin (fruit fly) showing the three exon regions with respective sequence length, 2005—RLPs synthesis by recombinant technology, 2009—domain selection and design of modular RLP, 2011 (top)—RLP (antigen) and RLP-antibody interaction for bioimaging, 2011 (bottom)—plot (temperature versus hydrodynamic diameter) showing both upper critical solution temperature (UCST) and lower critical solution temperature (LCST), dual-phase behaviour of RLPs, 2012—structural conformation of full-length resilin to stress, 2013—crosslinked network between RLPs (blue) and four-arm polyethylene glycol (PEG; green) hybrid hydrogel, 2015—plot (temperature versus ultra-violet absorbance) showing soluble to insoluble transition of RLPs in aqueous solution and 2018—resilin gene expression in plants.

The amino acid sequence of resilin was first identified in early 2000s from the CG15920 gene segment of the fruit fly *Drosophila melanogaster*, which opened up new opportunities for synthesis and development of biomimetic resilins^11^. The CG15920 gene comprises N-terminal (exon-1), C-terminal (exon-3) and the middle chitin-binding (exon-2) domains, where exon-1 and exon-3 consist of 18 and 11 copies of consensus amino acid sequences: GGRPSDSYGAPGGGN and GYSGGRPGGQDLG, respectively^11^. The first recombinant pro-resilin or resilin-like polypeptide (RLP), namely Rec1-resilin (encoded from the exon-1 of CG15920 gene) was synthesized in mid-2000s as a water soluble polypeptide expressed in the bacteria *Escherichia coli*^12^. The synthesized pro-resilin was photo-crosslinked (dityrosine) using a ruthenium-persulfate crosslinking system to form hydrogels, which exhibited 97% resilience, outperforming native resilin dissected from dragonfly tendon (92%), natural elastin (90%) and synthetic polybutadiene rubber (80%)^12^. However, the reported yield of RLPs was only 15 mg/L of culture, which was later optimized to more than 20-fold increase (300 mg/L of culture) by applying an improved lactose-induced fermentation method^13^. Over the last decade and a half, several other RLPs of different length, amino acid repeat sequences and insect genes were also synthesized using isopropyl β-d-1-thiogalactopyranoside (IPTG) induction and Studier autoinduction methods, purified by heat and salting-out and affinity chromatography techniques, and crosslinked with covalent and metal coordination crosslinks to form hydrogels.^14,15^ Figure 1 illustrates the timeline of key discoveries and milestones of resilins and RLP-based biomaterials.

The synthesized RLPs have several advantages over other elastomeric polypeptides, such as elastin-like polypeptides (ELPs), silk-like polypeptides (SLPs) and collagen-like polypeptides (CLPs)^1^, which are highlighted in Box 1 and discussed throughout the manuscript. However, the hydrogels fabricated from pristine RLPs showed no cellular attachment and proliferation, limiting their applications in tissue engineering^16^. This led to the design and synthesis of the first modular RLPs (with a yield of 80 mg/L of culture), and development of the modular RLP-based hydrogels with improved biological and structural properties in late 2000s^17^. Advances in the field of modular protein engineering^18^ and biofabrication^19^ have led to the design (via computational modelling), synthesis and development of a plethora of novel repurposed (different or modified from native function) modular protein-based biomaterials over the last two decades, which have gained increasing attention in tissue engineering, regenerative medicine, drug delivery and biosensor applications^20^. Structurally, RLPs have been identified as a class of unusual representative of the intrinsically disordered proteins (IDPs) that have changed the structure–function paradigm of proteins. In this review, we consider the central role of fundamental structure–property relationship and applications of RLPs, and discuss their evolution with design and development of modular RLP-based biomaterials, their biomedical applications, current challenges and future perspectives.

Box 1 Highlights **Advantages of RLPs over other elastomeric polypeptides**

- Unique sequence rich in uncharged, polar amino acids and devoid of canonical hydrophobic residues, and contains higher proportion of glycine- and proline-rich segments.
- Average negative hydropathy index.
- Intrinsically disordered protein structure with rapidly interchangeable conformational ensemble in physiological conditions.
- Multi-stimuli (pH, temperature, ions, mechanical stress, other molecules, etc.) responsiveness, including dual-phase transition behaviour (existence of both upper critical solution temperature, UCST and lower critical solution temperature, LCST).
- Low stiffness, high extensibility, outstanding resilience and excellent fatigue life.
- No inflammatory response.

**Key points for understanding and development of RLP-based smart systems**

- The self-assembly and responsive properties of RLPs are substantially influenced by their amino acid composition, average hydropathy index and length of repeat motifs.
- Sequence manipulations readily alter self-assembly and responsive properties of RLP-based systems, therefore, an attractive tool in biomedical engineering.
- Modular protein engineering offers an excellent toolbox for selection, design and development of physiochemically tuneable RLP-based biomaterials with desired properties and functions.
- Profiling sequence and stimuli responsiveness of RLPs can guide selection and programming of modular RLPs with desired structural, responsive and functional properties.
- Both quality (structure, hydrophobicity, stimuli responsiveness and mechanical properties) and quantity (molecular weight or length of repeat motifs) of secondary domains affect stimuli responsiveness of modular RLPs.

## Engineering structure, composition and stimuli responsiveness of resilin-mimetics

### Pristine RLPs

**Fig. 2:**
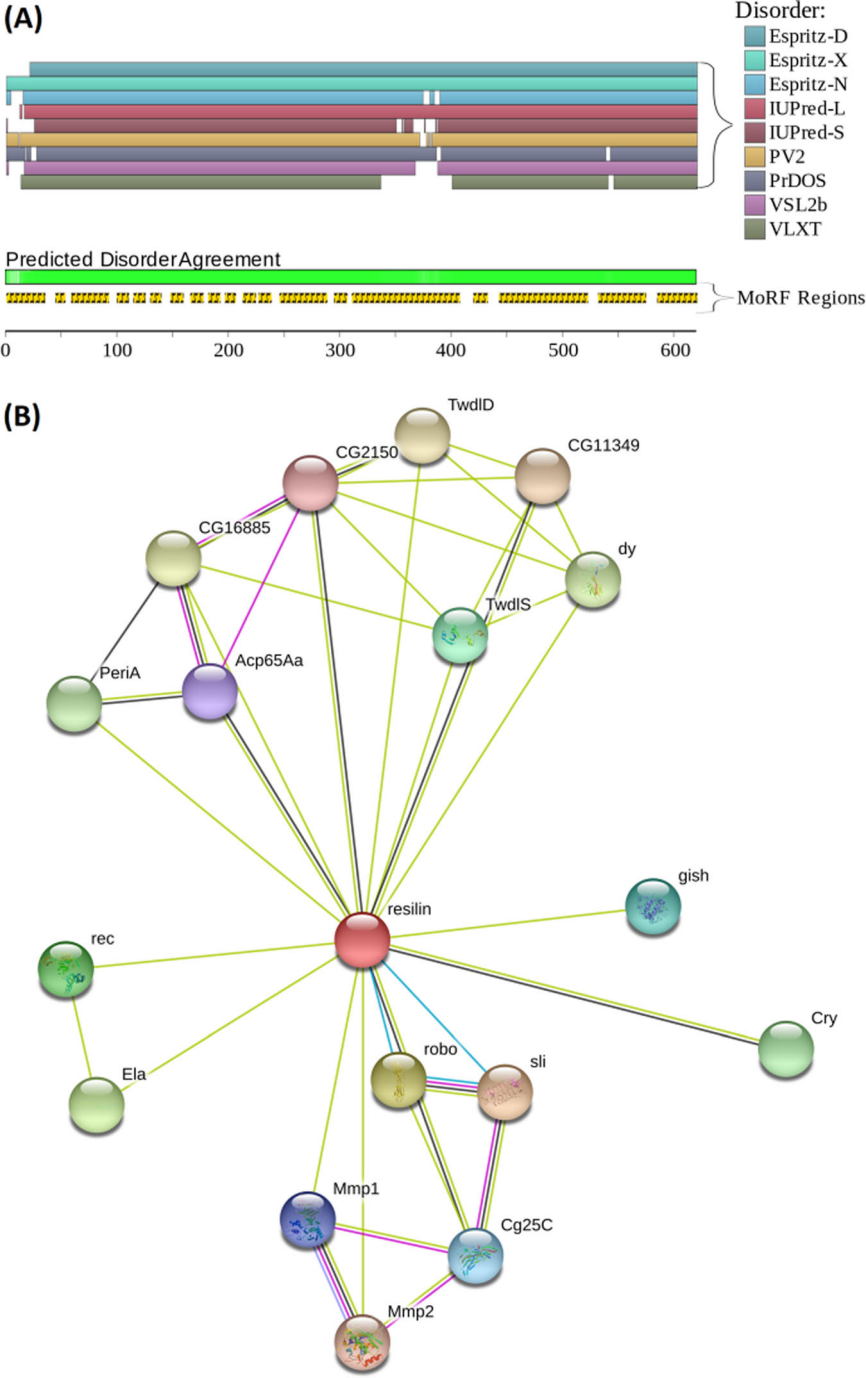
Evaluation of functional intrinsic disorder propensity and interactivity of *D. melanogaster* pro-resilin: full-length resilin (UniProt ID: Q9V7U0). **a** Functional disorder profile generated by D^2^P^2^ platform (http://d2p2.pro/)^26^. The nine different coloured bars represent location of disordered regions found by different disorder predictors (Espritz-D, Espritz-X, etc.). The green bar corresponds to disordered regions by consensus, and shows the predicted disorder agreement between these nine predictors. The yellow bar corresponds to disorder-based binding sites known as molecular recognition features (MoRFs). **b** Interactability of resilin analysed (using the medium confidence level of 0.4) by STRING platform (http://string-db.org/cgi/)^27^. The coloured nodes represent query protein (red) and first shell of protein interactors (with gene name or ID), where empty and filled nodes represent proteins of unknown 3D structure and some known or predicted 3D structure, respectively. Protein–protein interaction is represented by differently coloured lines, where green line represents neighbourhood evidence, black line represents co-expression evidence, purple line represents experimental evidence and light blue line represent database evidence.

Based on the amino acid sequence and composition, one can predict the structure of RLPs to fall under the natively unordered region in the Uversky plot (mean net charge versus mean hydrophobicity) of proteins (sourced from protein database), which correlates well with the amorphous nature of the protein determined by early structural investigations^5,21^. Subsequent experimental investigation of the secondary structure of RLPs using circular dichroism (CD), Fourier-transform infrared (FTIR) and nuclear magnetic resonance spectroscopy revealed their overall structure to be largely unordered (random coil secondary structure conformation) with possible coexistence of β-turns and polyproline II helix (PPII) conformations, and no apparent α-helical or β-sheet features^22–24^. Moreover, the RLPs exhibited no significant change in their secondary structure over a wide range of pH and temperatures in dilute aqueous solution^23,24^. However, when putative pro-resilin of 12 *Drosophila* species and few other insects were compared, the consensus sequence GGRPSDSYGAPGQGN was observed to be commonly present (8 out of 12) among the studied *Drosophila* genus, whereas significant difference, such as higher proline and lower glycine content and absence of chitin-binding domain, was observed for the mosquito (*Anopheles gambiae*) pro-resilin (consensus sequence GAPAQTPSSQY) that indicates possible structural and/or property differences of resilin among different insect genus^25^. Furthermore, the evaluation of resilins’ functional intrinsic disorder propensity (Fig. 2a) by D^2^P^2^ platform and interactivity (Fig. 2b) using STRING platform emphasizes their largely disordered nature; however, it also assessed the presence of a multitude of disorder-based binding sites that might define their interactivity and multifunctionality^26,27^. Over the last two decades, increasing evidence of unordered structure (from structural biology) with functional properties of IDPs has dramatically changed the paradigm that protein function depends on a fixed 3D structure^28^. Indeed, IDPs have been identified as a very large and functionally important class of proteins, which lack a fixed or ordered three-dimensional (3D) structure. In this context, the RLPs were experimentally established as IDPs with their conformational ensembles and molecular dynamics described using small-angle X-ray (SAXS)  and neutron scattering (SANS) techniques where the RLPs exhibited asymmetric distance distribution function as well as non-converging dimensionless Kratky plot, which are characteristics of IDPs^29–31^. The intrinsic disorder in RLPs arises from their low sequence complexity, repeat amino acid sequences containing a large fraction (>30%) of recurring structure breaking proline (P) and glycine (G) pairs (i.e. P-X_4_-G motifs, where *X* is any amino acid residue except P and G)^32^. The ability of largely unordered structure of RLPs to exhibit outstanding resilience when crosslinked and water swollen further motivated studies on their stimuli-responsive properties and self-assembly.

The capability of some proteins and polypeptides to change their conformations and self-assemble in response to one or more external stimuli, such as pH, temperature, ions, etc., is encoded in the amino acid sequence level through the types of interactions they are involved^33^, and at the macromolecule level through chain length^34^. The naturally occurring amino acids can undergo various types of interactions including covalent, electrostatic, steric, hydrophobic, π–π stacking and hydrogen bonding. The first endeavour to study the stimuli-responsive properties of RLPs at different pH was performed using quartz crystal microbalance with dissipation monitoring, where Rec1-resilin adsorbed at different orientations onto gold substrate depending on pH of the environment. The RLP exhibited back-on adsorption at pH 2, compact end-on bilayer adsorption at pH 4.9 and side-on adsorption at pH 12^35^. However, when pinned onto the gold surface at experimental isoelectric point (IEP) of pH 4.9 (measured using dynamic light scattering technique), Rec1-resilin was able to switch (kinetically fast, robust and completely reversible) conformation from a compact mushroom-like (at IEP) to brush-like (IEP < pH <10.5) to an extended brush-like (pH > 10.5) conformation, thereby changing viscoelasticity and packing density^35^. Moreover, the amino acid sequence of Rec1-resilin exhibited discrepancy in experimental and theoretical IEP (pH 9.2), which indicates complex organization of RLPs in aqueous solutions. Rec1-resilin’s amino acid sequence consists of significant polar, acidic and basic residues (aspartic acid, glutamic acid, lysine and arginine), which contribute to negative zeta potential (hydrophobic residues buried) in aqueous solutions above experimental IEP and vice versa^36^. Simultaneously, the intrinsic photo-responsive property of RLPs, which arises from the tyrosine residues (excitation/emission, Ex/Em at 275/304 nm) present in their repeat sequence, was also observed to be sensitive to pH (reversibly forms tyrosinase with Ex/Em at 295/345 nm at pH > 10.5) and surface plasmon resonance of metal nanoparticles (NPs) in near vicinity^36,37^.

Interestingly, careful examination of the change in the hydrodynamic diameter of Rec1-resilin in aqueous medium showed dual-phase transition behaviour (DPTB), i.e., existence of both upper critical solution temperature (UCST) at around 6 °C and a lower critical solution temperature (LCST) at around 70 °C, which is quite rare^36^. The DPTB was also observed in other RLPs, namely An16 (encoded from mosquito BX619161 gene), which confirms the unusual temperature-responsive properties of RLPs across different insect genus^29^. Unlike ELPs, which are enriched in nonpolar amino acid residues (positive hydropathy index) and exhibit only LCST^38^, RLPs are enriched in polar residues (negative hydropathy index) and exhibit DPTB^36^. Moreover, the LCST of RLPs was observed to be kinetically controlled with hydrophobic aggregations, whereas UCST to be controlled by pH^29,36^. In addition, ions/kosmotropes were observed to modulate the critical solution temperatures of RLPs following the Hofmeister series, where divalent ions have a strong effect compared to monovalent ions^29,39^. Based on the in-depth sequence heuristics study to encode the dual-phase behaviour of RLPs in the amino acid sequence level, the LCST behaviour of RLPs is predicted to arise from nonpolar amino acid residues and P-X_*n*_-G motifs, whereas the UCST behaviour from zwitterionic motifs and aromatic amino acids^32^. However, for polypeptide with zwitterionic motifs and no aromatic amino acids, no phase behaviour was observed at physiological pH, whereas UCST was observed at acidic pH^32^. In a separate study, when tyrosine (Y) residues were replaced by phenylalanine (F) and methionine (M) in the primary structure, the RLPs did not exhibit UCST behaviour^39^. Furthermore, with negative zeta potential measured at neutral pH and lack of phase behaviour at pH 12 for Rec1-resilin, it is evident that additional residue interactions, such as cation–π interactions (between arginine (R) and aromatic residues) significantly influence UCST^32,36^. The UCST has been observed to increase with cation–π interactions in the order of tryptophan (W) >> tyrosine (Y) > phenylalanine (F) >> histidine (H)^34^. In addition, the dynamics of phase separation of polypeptides were also observed to be controlled by design parameters, such as increase in the ratio of aromatic:aliphatic residues, RLP repeats or length or molecular weight, and average hydropathy index (AHI) increases UCST^32,34^. Conversely, the UCST behaviour exhibited by An16, which does not contain the zwitterionic pattern like Rec1-resilin and lysine/arginine (for cation–π interaction), with no change in zeta potential is quite intriguing and needs further systematic investigation^29^. Based on the sequence heuristics study, the DPTB of RLPs with consensus repeat sequence GGRPSDSYGAPGGGN is presumed to stem from the fusion of a UCST motif (GGRPSDSYG) and a putative LCST motif (APGGGN)^32^.

On the other hand, the RLP, namely RES50 containing the pentapeptide sequence (PGGGN)_10_ and exhibiting predominantly random coil secondary structure, showed tendency to self-assemble into micron-long fibres (50–80 nm diameter, measured using atomic force microscopy (AFM)) when equilibrated at ambient temperature in water^24^. The self-assembled fibre structure was dissimilar to both elastin- and amyloid-like fibres, and showed compact tubular structure without any propensity for twisting^24^. Conversely, Rec1-resilin and An16 showed no tendency to form fibres. However, when RLP, Rec1-resilin was equilibrated in the presence of silk fibroin (SF) molecules, it not only triggered self-assembly of co-assembled rod-like structures but also shifted the LCST of co-assembled structures to physiologically relevant temperatures^40^. The occurrence of pH, temperature, light, ion and other macromolecule responsiveness in a single molecule makes the RLPs a versatile multi-stimuli-responsive material. The rapid increase in our understanding of amino acid sequence, structure, stimuli responsiveness and self-assembly of pristine RLPs will expand our knowledge and ideas on design, development and application of new programmable protein-engineered modular RLPs.

### Modular RLPs

**Fig. 3:**
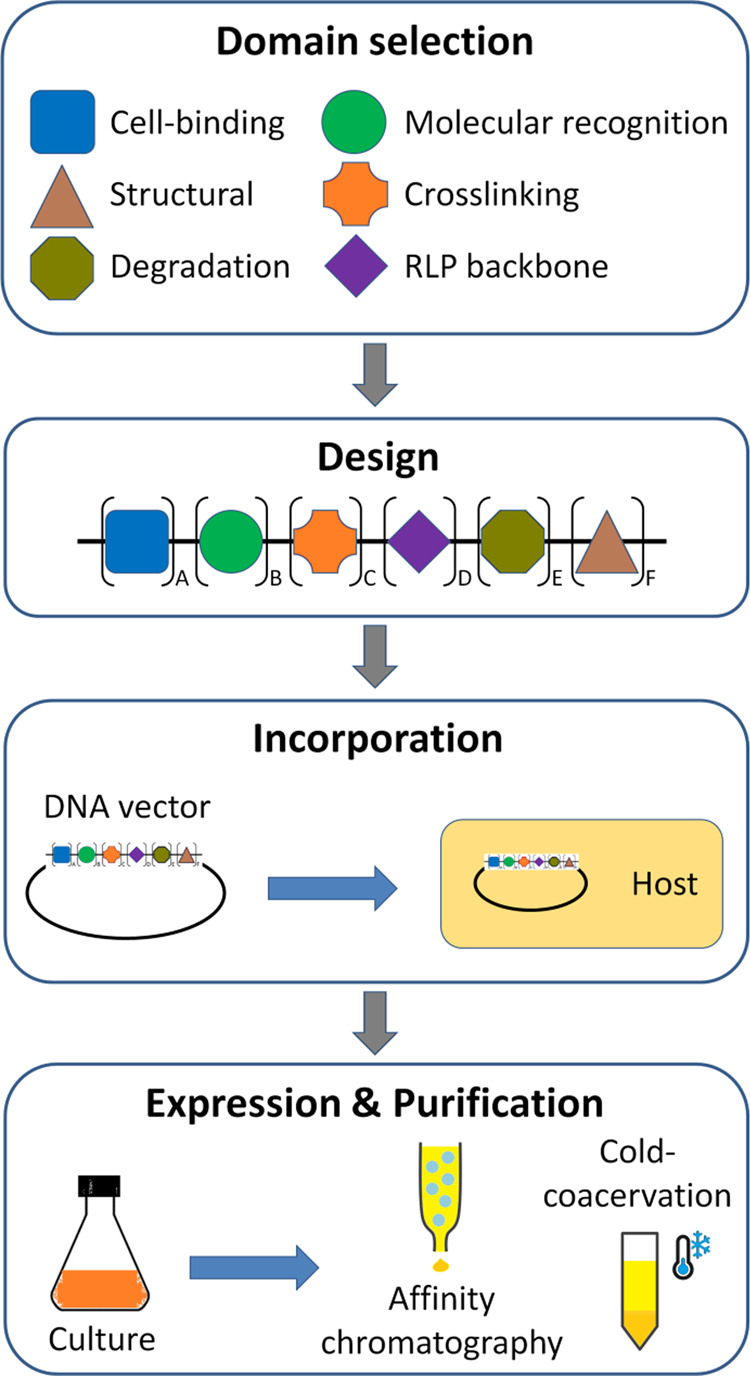
Schematic of key steps involved in modular RLPs synthesis. The domain selection step involves the selection of peptide domains of desired functions, the design step involves the arrangement of the intended amino acid sequence and encoding genetic sequence, the incorporation step involves the construction of a genetic vector and their transfection into a host organism, the expression involves induced expression of the intended modular RLPs in host organism, and purification step involves extraction of pure polypeptides by chromatographic and/or non-chromatographic (cold-coacervation) methods.

Modular RLPs are obtained by appropriate selection of secondary domains (structural and functional), their design with the RLP backbone incorporation, expression and purification of engineered multifunctional amino acid sequence (Fig. 3). The overall structure, composition, stimuli responsiveness and self-assembly properties of modular RLPs may vary from the pristine RLPs based on the secondary domains. The first synthesized modular RLP—namely RLP_12_-RGD_2_-MMP-HBD—comprising RLP encoded from exon-1 of *D. melanogaster* gene, cell-binding sequence (RGD), matrix metalloproteinase cleavable peptide (MMP) and heparin-binding domain (HBD), showed predominantly random coil conformation with a small fraction of β-turns (measured using CD spectroscopy)^17^, similar to that of pristine RLPs^23,24^. On the other hand, when a trimeric modular RLP, namely REC, or RLP_4_-ELP_7_-CLP_2_ was synthesized and investigated for responsive properties, the modular RLP exhibited predominantly PPII conformation and self-assembled into network of flexible aligned fibres (different from that of the twisted rope-helical structures observed for some glycine-rich ELPs) in water^41^, which is similar to that of RES50, and needs further investigation^24^. Moreover, the self-assembled RLP_4_-ELP_7_-CLP_2_ fibres exhibited high tendency in bending with Young’s modulus in the range of 0.1–3 MPa, which is lower than those observed for usually straight and stiff amyloid-like fibres (in the range of tens of GPa)^41^. In a separate study, a suite of new modular RLPs, namely An_*X*_-EGFP (where *X* = 4, 8, 16 or 32)—comprising RLP (An_*X*_) encoded from *A. gambiae* gene and enhanced green fluorescence protein (EGFP), exhibiting green fluorescence when excited with 485 nm light, were synthesized (with yield in the range of 14–115 mg/L of culture)^42^. Such modular RLPs have potential to find applications in bioimaging^43^. Another modular RLP—namely RZ_10_-RGD—comprising RLP (RZ_10_) encoded from *A. gambiae* gene was also synthesized (with yield around 22 mg/L of culture) and applied in the field of tissue engineering^44^. However, no significant difference in their secondary structure was observed. Conversely, when the modular RLP—namely RLP_4_-SLP_4_—comprising RLP (as soft block) encoded from exon-1 of *D. melanogaster* gene and SLP (as hard block) was synthesized and investigated for stimuli-responsive properties, the modular RLPs formed spherical micelles (10–20 nm diameter with SLP core) at 4 °C, which readily transformed to nanofibrillar structure (80–200 nm length) at 37 °C and large coalescent nanostructures at 70 °C (observed using AFM)^45^. Although the mechanisms and molecular events of self-assembly are unclear at this point, it is presumed that the interaction (weak forces, such as hydrophobic interaction, electrostatic interaction and hydrogen bonds) between resilin and silk blocks could have played a major role in overcoming the propensity of nanofibril (β-sheet structure) formation by the silk blocks and directing the self-assembly^45^. As a step further, to understand the effects of RLP chain length on the UCST and the LCST behaviour and the mechanical properties of RLP–SLP block co-polypeptides, modular RLPs, namely RLP_4_-SLP and RLP_8_-SLP, were synthesized and investigated for thermal responsiveness. These modular RLPs revealed an upshifted UCST and stiffness of the reversible hydrogel with increase in RLP chain length, whereas differentially affected LCST^46^. In addition, the modular RLPs were also observed to be pH and ion responsive, where moderate concentrations of potassium phosphate and sodium chloride downregulated both the UCST and LCST and hydrogel mechanical properties^46^.

**Fig. 4:**
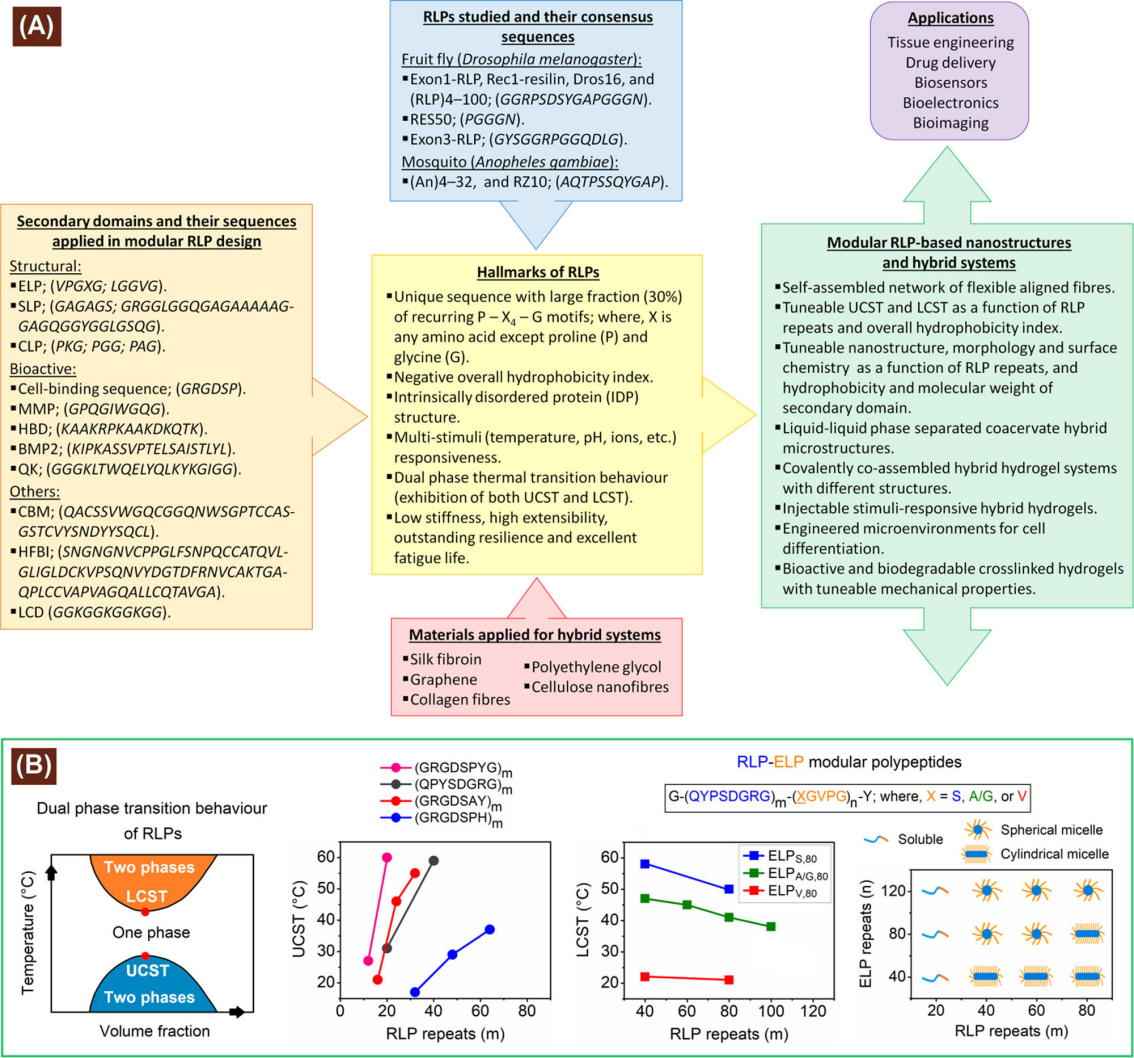
Hallmarks of RLPs, development of modular RLP-based materials and their structure–property relationship. **a** Summary of RLPs, secondary domains and other materials applied for development of modular RLP-based nanostructures and hybrids. The amino acid sequences of RLP repeats and secondary domains are presented as a single letter code. UCST upper critical solution temperature, LCST lower critical solution temperature, ELP elastin-like polypeptide, SLP silk-like polypeptide, CLP collagen-like polypeptide, MMP matrix metalloproteinase cleavable peptide, HBD heparin-binding domain, BMP bone morphogenetic peptide, QK vascular endothelial growth factor-mimicking domain, CBM cellulose-binding module, HFBI hydrophobin protein domain and LCD lysine crosslinking domain. **b** Schematics of dual-phase transition behaviour and some of the structure–property relationship of RLP- and modular RLP-based systems^34,48^.

The highly hydrophobic ELPs containing around 95% proline and glycine content combined have been reported to influence the self-assembly of other polypeptides, including SLPs resulting in various nanostructures, such as NPs, nanofibres, nanogels, etc.^47^ In order to study the influence of ELPs on the LCST and self-assembling properties of modular RLPs, a subset of modular RLPs, namely RLP_*n*_-(X-ELP)_*Y*_ (where *n* = 20, 40, 60, 80 and 100; *Y* = 40, 80 and 160; *X* is amino acid residues—serine, valine and equal content of alanine/glycine) were synthesized and examined for their stimuli responsiveness^48^. The modular RLP, namely RLP_40_-(S-ELP)_80_ formed spherical micelles (RLP core) at 35 °C (UCST), coacervates (colloidal droplets) at 58 °C (LCST), whereas soluble chains in between these temperatures (observed using cryo-transmission electron microscopy, cryo-TEM)^48^. The RLP block length, ELP block length and AHI were observed to be the key parameters to tune self-assembly (morphology) and LCST of modular RLP–ELP block co-polypeptides, where increasing the RLP chain length, decreasing ELP chain length and increasing AHI drove spherical to cylindrical micelle morphology transition. In addition, increasing AHI decreased LCST^48^. Furthermore, in order to study the effect of different terminal domain on coacervate size and structure, two modular RLPs—namely CBM_2_-RLP-HFBI and CBM-RLP-CBM—comprising cellulose-binding module (CBM) and amphiphilic hydrophobin protein domain (HFBI) were synthesized and investigated^49^. The salt-induced coacervate size of CBM_2_-RLP-HFBI was observed to be larger than CBM-RLP-CBM (observed using cryo-TEM) due to hydrophobic interactions of HFBI, and tuneable with varying protein concentration, temperature and pH^49^. The modular RLPs developed so far have great potential for controlled release, drug delivery, biosensor and injectable hydrogel applications. Figure 4 illustrates the hallmarks of RLPs, development of modular RLP-based systems and their structure–property relationship. A summary of modular RLPs (that employ the concept of utilizing peptide domains for repurposed functionalities) synthesized, their structure and property transformations, and respective biomedical applications are presented in Table 1. Thus, significant knowledge has been developed with RLPs of sequence-definable modular structure and properties, which are a prerequisite for design of future materials with inherent bio-functionality of naturally derived materials and the tunability of structural biology. Such foundation certainly paves the way for development of high-yield expression of modular RLPs, and scaling up protein expression from bench-top to a production facility.

**Table 1:** Summary of modular RLPs synthesized and their reported structure–property transformations.

Modular RLPs^a^	Secondary domains	Structure–property transformations	Applications	Ref.
RLP_12_-RGD_2_-MMP-HBD, RLP_12_-RGD, RLP_12_-HBD, RLP_12_-MMP	Cell-binding sequence (RGD), matrix metalloproteinase cleavable peptide (MMP), heparin-binding domain (HBD)	Increase in cellular adhesion, biodegradation and metabolic activity (with no inflammatory response), and mechanical property	Tissue engineering	^17,63,64^
REC or RLP_4_-ELP_7_-CLP_2_	Elastin-like polypeptide (ELP), collagen-like polypeptide (CLP)	Self-assembled network of flexible often aligned fibres (different to that of the twisted rope-helical structure) at physiological temperature	Tissue engineering	^41^
An_X_-EGFP (where *X* = 4, 8, 16 and 32)	Enhanced green fluorescence protein (EGFP)	Autofluorescence.	Bioimaging	^42^
RZ_10_-RGD, RZ_10_-BMP2, RZ_10_-QK	Cell-binding sequence (RGD), bone morphogenetic peptide (BMP), vascular endothelial growth factor-mimicking domain (QK)	Increase in cellular adhesion and differentiation, and mechanical property	Tissue engineering	^44,67–70^
RLP_*n*_-SLP_*n*_ (where *n* = 1, 4 and 8)	Silk-like polypeptide (SLP)	Spherical micelle (SLP core) to nanofibril to large coalescence with increase in temperature. Upshift in UCST and stiffness of hydrogel with increase in RLP length. Increase in cellular adhesion and mechanical property	Drug delivery, tissue engineering	^45,46^
(RLP)_*n*_-(X-ELP)_Y_ (where n = 20, 40, 60, 80 and 100; _Y_ = 40, 80 and 160; *X* is amino acid residue S, A/G (equal content) and V	Elastin-like polypeptide (ELP)	Spherical micelle (RLP core) to soluble chains to coalescence with increase in temperature. Spherical to cylindrical morphology transition with increase in RLP chain length and hydrophobicity of the ELP	Drug delivery, tissue engineering	^48^
CBM_2_-RLP-HFBI, CBM-RLP-CBM	Cellulose-binding module (CBM), hydrophobin protein domain (HFBI)	Increase in coacervate size with increase in temperature and pH. Selective adhesion and self-assembly on different surfaces	Tissue engineering, drug delivery, biosensor	^49,56^
GB1-RLP_4_, GB1-RLP-(GB1)_5_-RLP-(GB1)_4_-RLP	Artificial elastomeric protein (GB1)	Increase in mechanical property	Tissue engineering	^66^
RLP_12_-LCD_5_	Lysine crosslinking domain (LCD)	Increase in lysine crosslinking site and mechanical property	Tissue engineering	^75^

^a^The RLPs, namely An_*X*_ and RZ_10_ are constructed using the mosquito BX619161 gene, whereas the rest using the fruit fly CG15920 gene.

### Guided self-assembly and multifunctional soft-templating using resilin-mimetics

The first endeavour to study the directed self-assembly of RLPs was performed on different material surfaces, such as mica, silicon wafer and highly ordered pyrolytic graphite and was observed using AFM. The experimental RLP, Rec1-resilin exhibited substrate surface energy-dependent conformation and self-assembly, forming a columnar structure on hydrophobic surface and an energetically favourable monomolecular layer structure on the hydrophilic surfaces^50^. Shortly after, the ability of RLPs to generate and/or stabilize inorganic nanomaterials using biomimetic approach was also investigated to expand its potential in nanomaterials engineering applications. In this approach, RLPs are incubated with the metal ions in aqueous solution under a controlled environment (pH, temperature), for specific period of time, followed by reduction of the metal precursor using an exogenous reductant to convert the metal ions to zero-valent metal atoms. Rec1-resilin was the first RLP to be used as a soft molecular template to direct stabilization of optically coupled hybrid architectures of noble metal nanoparticles (NPs)^37,51^. The borohydride reduced gold (Au) and platinum (Pt) NPs were stabilized in the size range of 1.8–20.0 nm (observed using TEM) using the pH responsiveness of the Rec1-resilin. The growth of the crystal phase in this bio-mineralization process is dictated by the crystal phase-peptide segment interaction. The peptide caps the growing NP on the surface, manipulating crystal growth direction, arresting overall particle growth, stabilizing the particle in colloidal suspension and generating the bio-interface. Unlike thiol-mediated stabilization reported for globular proteins and synthetic polymers, Rec1-resilin (containing no sulfur containing amino acid residues) stabilized the NPs by non-covalent mode of binding through the collective interactions of the amino acid residues, which made the surface of the NPs effectively available for chemical reactions^37,51^. Recently, the RLP-directed PtNPs were also loaded on to carbon supports, and successfully demonstrated for fuel cell electrocatalytic applications^51,52^. Furthermore, the pH responsiveness of the RLPs was also harnessed to generate fluorescent metal sub-nanoclusters (FMNCs), where the deprotonation of tyrosine residues in RLPs (at pH >10.5) led to electron release for reduction of metal ions, and the RLPs acted simultaneously as the directing agent, chemical reducer and highly efficient stabilizer (i.e. one-pot approach)^53–55^. These FMNCs are composed of a few to a hundred atoms and their sizes are comparable to the Fermi wavelength of electrons, resulting in molecule-like properties including discrete electronic states and size-dependent fluorescence. Facile synthesis of such fluorescent metal NCs with tuneable emission colours has potential to establish them as a new class of ultrasmall, biocompatible fluorophores for applications as biological labels or optoelectronic emitters.

Lately, advancement in directed self-assembly of modular RLP-based systems was performed using CBM_2_-RLP-HFBI and CBM-RLP-CBM, where dip-coated CBM_2_-RLP-HFBI coacervate adhered as non-continuous layer to both cellulose and graphene surfaces, whereas CBM-RLP-CBM coacervate adhered as separate micro-sized particles to only cellulose surface (observed using AFM)^49^. In addition, mechanical extension experiments performed at different pH (5 and 11) using CBM_2_-RLP-HFBI by adhering CBM_2_ end to a thin layer of cellulose on one side and HFBI end to octadecyl trichlorosilane (OTS)-coated AFM tip on the other side showed directed or force-induced conformational stretching of the RLP chain^56^. A single molecular stretching of 6% was observed for the modular RLP at pH 5 with random coil conformation, which could not be fully extended by tensile stress, whereas 11% stretching was observed at pH 11 with swollen coil conformation, which could be fully extended by tensile stress^56^. Such directed responsiveness and self-assembly of RLPs have potential to find applications in biosensing and soft-robotics. As the library of functional polypeptide domains continues to expand, by using rational protein engineering design, it is possible to modulate amino acid sequences to gain control over a range of functional properties of modular RLPs for desired applications. Some key points for understanding and development of RLP-based programmable systems are highlighted in Box 1.

### Mechanism of elasticity and chemical crosslinking of RLPs

The elastomeric functions of self-organizing and self-assembling proteins are dependent on conformational disorder and hydration, which is apparent above a threshold in proline (P) (which interrupts the transmission of stable secondary structure) and glycine (G) (which engenders flexibility) content and composition in the primary structure^57^. The predicted capacity of RLPs is well above the proline–glycine threshold^57^, and the elastic properties of RLPs depend on the polarity of the side chains, where an increased polarity showed higher extensibility and lower stiffness (calculated using molecular dynamic simulations)^58^. The polar groups of naturally occurring motif form strong hydrogen bonding with surrounding water molecules that act as deformation energy-absorbing layer, thereby improving elasticity, whereas the hydrogen bonds formed on the backbone of the protein showed only minimal effects on the extensibility or stiffness^58^. Moreover, photo-crosslinked RLP hydrogels exhibited higher molecular chain mobility and viscoelasticity (measured using differential scanning calorimetry, DSC and ultra-microindentation techniques) with increase in level of hydration^59^. In a full-length resilin produced from the three exons of *Drosophila* CG15920 gene, exon-1 yields the ‘soft’ segment with more hydrophilic blocks and exhibits >90% resilience, whereas exon-3 yields the ‘hard’ segment with a large hydrophobic block in the middle and exhibits resilience around 63% (measured using AFM)^60^. However, when full-length resilin and exon-3 encoded polypeptide films were stretched up to 1.5 times of the original length, increase in β-turn structure was observed for both of them (measured using FTIR spectroscopy), whereas no significant change was observed for exon-1 encoded polypeptide^61^. It has been proposed that the exon-1 encoded polypeptide absorbs the mechanical energy and remains unstructured, whereas the exon-3-encoded polypeptide receives energy from exon-1-encoded polypeptide and transforms to an ordered β-turn structure to store energy. Once the stress is removed, the exon-3-encoded polypeptide undergoes reversible structural transformation and transfers the absorbed energy to exon-1-encoded polypeptide^61^. Therefore, it is hypothesized that the full-length resilin acts as hydrophilic–hydrophobic–hydrophilic block-copolymer, which forms irregular-sized micelle structure (directed by hydrophobic block) upon hydration and β-turn structure upon stretching^61^.

Over the past years, a variety of crosslinking strategies have been successfully applied to fabricate covalently crosslinked pristine RLP hydrogels, which can be potentially extended to fabricate RLP-based modular hydrogels^14,15^. Ruthenium-persulfate-mediated photochemical crosslinking is a popular method for RLP-based hydrogel fabrication due to its fast reaction time (<2 min), biocompatibility and formation of dityrosine crosslinks that mimic natural resilin in insects^12^. This method can also be potentially applied for coating and crosslinking RLPs to surface phenolic group functionalized material surfaces to control cellular adhesions^16^. In addition, double network structures with improved mechanical properties can also be achieved in photo-crosslinked hydrogel systems using mutant RLPs (engineered with histidine amino acid residues) and addition of Zn^2+^ ions to obtain the second metal coordination crosslinks^62^. Interestingly, metalloenzyme-mediated chemical crosslinking of tyrosine groups using horseradish peroxidase and citrate-modified photo-Fenton system produces dityrosine crosslinked RLP hydrogels and/or 3,4-dihydroxy-dl-phenylalanine (DOPA) moieties, which have potentials for fabricating RLP-based sticky hydrogels^60^. Furthermore, Mannich-type condensation reaction using tris(hydroxymethyl)phosphine (THP), which crosslinks primary amines of lysine residue in RLPs with hydroxymethylphosphine group in the crosslinkers, can also be employed to form hydrogels^63^. The advances in our knowledge and understanding of the molecular mechanism of pristine resilin elasticity and various crosslinking strategies will expand our ideas on development of new modular RLP-based hydrogels with functional properties, such as stickiness and self-healing, and crosslinked networks with tuneable mechanical properties.

## RLP-based modular hydrogels: bioactivity, tuneable mechanical properties and biomedical applications

### Modular RLP hydrogels

The first modular RLP, RLP_12_-RGD_2_-MMP-HBD, was crosslinked to form hydrogels via the Mannich-type reaction using the crosslinker [tris(hydroxymethyl)phosphino]-propionic acid. This crosslinked modular RLP hydrogels exhibited good NIH-3T3 mouse fibroblast cell attachment and metabolic activity^17^, and tuneable elastic modulus in the range of 0.2–2.5 kPa, which has great potential for vocal fold tissue engineering applications^64^. Subsequently, the mechanical property and biological activity of hydrogels fabricated from a suite of modular RLPs, namely RLP_12_-RGD, RLP_12_-HBD and RLP_12_-MMP, and their combinations were investigated to examine the effects of the quality of the secondary domains^63^. The pristine RLP_12_ hydrogel displayed no cellular adhesion with round human mesenchymal stem cell (hMSCs) morphology, whereas the RGD and HBD containing modular RLP hydrogels displayed good cellular adhesion and proliferation with elongated cell morphology, which is suitable for tissue engineering applications. In addition, the RLP_12_-MMP hydrogel exhibited approximate biodegradation half-life of 20 h^63^. Moreover, when subcutaneously transplanted in a rat model, the modular RLP hydrogels showed no inflammatory response^65^. In order to improve the mechanical property of RLP-based hydrogels, two modular RLPs—namely GB1-RLP_4_ and GB1-RLP-(GB1)_5_-RLP-(GB1)_4_-RLP—comprising GB1 polyprotein (an artificial elastomeric protein) were synthesized and photo-crosslinked to obtain unique mechanical properties (combining strength, extensibility and resilience) close to that of muscles^66^. The fabricated hydrogels exhibited Young’s modulus in the range of 50–70 kPa at 15% strain (which is close to that of myofibrils/myocytes, 60–100 kPa) and extensibility of ~135%, which can be potentially applied as tough hydrogels for tissue engineering^66^. In a separate study, the modular RLP, namely RLP_4_-SLP_4_, when photo-crosslinked exhibited hydrogel elastic modulus of 2.9 kPa, which increased to 7.0 kPa after preincubation at 37 °C for 4 h and photo-crosslinked^45^. The fabricated modular hydrogels showed good MC-3T3 mouse preosteoblast cell attachment and proliferation, suitable for soft tissue engineering^45^. On the other hand, a suite of modular RLPs—namely RZ_10_-RGD^44^, RZ_10_-BMP2 (ref. ^67^) and RZ_10_-QK^68^—comprising bone morphogenetic peptide (BMP2) and vascular endothelial growth factor-mimicking domain (QK) were synthesized, crosslinked and investigated for their cellular differentiation, mechanical property and degradation characteristics. The RZ_10_-RGD hydrogels were fabricated using THP^44^ and/or transglutaminase enzyme (TGase)^69^ as the crosslinker, which exhibited shear storage modulus in the range of 7–10 kPa, and excellent hMSCs viability and proliferation. Moreover, hydrogels fabricated from RZ_10_-BMP2 showed increased levels of alkaline phosphatase activity, calcium deposition and expression of bone-related genes; however, they did not synergize with the RGD within the context of modular RLP^67^. Conversely, hydrogels fabricated from RZ_10_-QK showed increased endothelial-specific markers and endothelial function and suggested that protein-engineered microenvironments are sufficient to promote endothelial differentiation in the absence of exogenous growth factors^68^. Recently, RZ_10_-RGD hydrogels were also fabricated using a redox-responsive crosslinker, 3,3′-dithiobis(sulfosuccinimidyl propionate) (DTSSP), which showed excellent NIH-3T3 fibroblast viability and proliferation, and potential in vitro degradation and drug (dextran) delivery applications^70^. However, the modular RLP hydrogels crosslinked with DTSSP exhibited shear storage modulus lower than the hydrogels crosslinked using THP^44^ and TGase^69^.

### Hybrid hydrogels

The hybrid hydrogels have potential to offer combined benefits of improved structural, functional and biological properties over their individual components. The first modular RLP-based hybrid hydrogels were fabricated by the Michael-type addition reaction involving crosslinking between cysteine (C) residues on the modular RLPs, RLP_*X*_-RGD_2_-MMP-HBD (where *X* = 12, 24, 36 and 48) and the terminal vinyl sulfone functional groups of synthetic 4-arm star polyethylene glycol (PEG) crosslinker. The hybrid hydrogels exhibited elastic moduli in the range of 5–10 kPa, and showed successful encapsulation and spreading of human aortic adventitial fibroblasts in 7 days, which has great potential for cardiovascular applications^71^. In addition, RLP_24_-RGD_2_-MMP-HBD-based hybrid hydrogel showed successful encapsulation and viable 3D culture of hMSCs, and tuneable strain to break and resilience in the range of 68–173% and 90–98% (at 20% strain), respectively^72^. Subsequently, RLP-based hybrid hydrogel fibres, namely RLP-CF fibres, were fabricated by crosslinking elastic RLP and stiff collagen fibres (CF) using 4-arm PEG-ether tetrasuccinimidyl glutarate as crosslinker, which interacts with amine groups of both proteins^73^. The hybrid fibres showed improved biomechanical properties, and human adult fibroblast cell attachment and proliferation^73^. In a separate study, the RLP-based hybrid hydrogel, namely RLP-SF was fabricated by ruthenium-mediated photo-crosslinking of tyrosine residues between the resilin and silk fibroin molecules (i.e. co-crosslinking), which combines highly elastic soft phase of resilin and hard phase of silk and significantly increased the tensile storage modulus of the RLP hydrogel^74^. Conversely, hybrid hydrogels crosslinking the Norbornene functionalized (with lysine residue) modular RLP, namely RLP_12_-LCD_5_—comprising lysine crosslinkable domains (LCD), and thiol-functionalized 4-arm PEG—were fabricated via a thiol–ene photoclick reaction using the photoinitiator lithium phenyl-2,4,6-trimethyl benzoylphosphinate (LAP)^75^. The fabricated hybrid hydrogels exhibited shear storage modulus in the range of 0.4–3.5 kPa, and showed viable 3D encapsulation and 2D spreading of hMSCs, which has potential applications in soft tissue engineering^75^. Lately, liquid–liquid phase separation (at 25 °C) followed by crosslinking has been developed as a promising method for fabricating heterogeneous microstructured modular RLP-based hybrid hydrogels^76,77^. Two variants of the system RLP_12_-LCD_5_: (i) non-functionalized and (ii) acrylamide-functionalized (with lysine residue) were fabricated and used. The lysine residues of the non-functionalized modular RLPs were crosslinked with amine end groups of 4-arm PEG using THP as a crosslinker^76^, whereas acrylamide-functionalized modular RLPs were crosslinked with 4-arm PEG-acrylate using LAP as a photoinitiator (under UV light)^77^. The size of the fabricated microgels was observed in the order of 10–90 μm, where the LAP-mediated crosslinked hydrogels exhibited superior shear mechanical properties, excellent hMSCs viability and proliferation, which has a great potential for drug delivery and injectable hydrogel applications^76,77^. Furthermore, co-assembled hybrid hydrogels made of acrylamide-functionalized RLP_12_-LCD_5_ and cysteine residue-containing peptide amphiphile (E3CY) were fabricated by thiol–Michael addition reaction (by incubating at basic pH) and/or thiol–ene photoclick reaction (using the photoinitiator LAP)^78^. The structure of hybrid hydrogels was tuneable in the form of nanofibres, beaded strings and nanospheres, which has potential to find applications in drug delivery, microfluidics and tissue engineering^78^. Recently, thiol–Michael addition reaction has also been applied to crosslink acrylamide-functionalized RLP_12_-RGD_2_-MMP-HBD and thiol-functionalized hyaluronic acid (HA) to form hybrid hydrogels^79^. In addition, physically crosslinked hybrid hydrogels formed via electrostatic interactions between the positively charged lysine residues of the RLP and the negatively charged HA backbone were also reported^79^. The fabricated hybrid hydrogels resembled mechanical properties of native vocal fold tissue, and were successfully demonstrated as a carrier for human bone marrow mesenchymal stem cells, with confirmed in vivo acute biocompatibility of injected hydrogels up to 3 weeks with only mild inflammation in vocal fold lamina propria^79^. In a separate study, CBM-RLP-CBM was employed as a pH responsive crosslinker for cellulose nanofibres (CNF), which increased the rigidity of the CNF hydrogel matrix^80^. Recently, multifunctional RLP_64_-graphene composite hydrogel was fabricated via carbodiimide (EDC) and *N*-hydroxysuccinimide (NHS)-mediated polymerization (amide bond formation between carboxylic acid and primary amine groups). The composite hydrogels demonstrated adhesion strength and electrical conductivity of 24 kPa and 0.9 S/m, respectively, along with 20% increase in stiffness and toughness, which has great potential for wearable sensor applications^81^. The developed modular RLPs and their pristine and hybrid crosslinked hydrogels have shown potential applications in many areas including, tissue engineering, drug delivery, bioimaging, biosensors and bioelectronics.

### Future perspectives

Protein- and peptide-based self-assembling responsive biomaterials hold unprecedented promise to facilitate development of functional materials for a variety of applications in human healthcare by preventing, delaying or reversing many disease pathologies based on innovative research. IDPs, in general, play key biological roles including regulation of cellular transcription, translation and signalling. They also play a central role in the ordered assembly of macromolecular machines such as the ribosome, in organization of chromatin, in assembly and disassembly of microfilaments and microtubules^28,82^. Moreover, IDPs containing low-complexity sequences can promote phase separation to form membrane-less organelles within the cytoplasm or nucleoplasm, thus contributing to their compartmentalization in a regulated manner^28,82^. Examples of functional IDPs also include HIV-1^83^ and COVID-19^84^ virus proteins that use the intrinsic disordered region as flexible armour for survival as well as weapon for host invasion. RLPs, being IDPs and displaying multi-stimuli responsiveness including dual-phase behaviour, multiplicity of their conformational ensemble created unmatched new opportunities. The rational protein engineering design with expanding library of peptide domains along with artificial intelligence, machine learning and advanced computational sequence heuristics approach represents unparalleled potential to develop smart RLPs with complex but controlled functionalities. Such advances also offer remarkable opportunity for development of new modular RLP-based biomaterials with desired bioactivity, crosslinking, mechanical property, self-healing, multi-responsive capability and selective biodegradation, which presents a multifunctional platform for increasing the efficacy of RLPs for specific biomedical applications. In this context, indeed, the rational design and development of new modular RLPs comprising several new motifs, such as structural domains from abductin, mussel adhesive protein, etc.^85,86^, cell-binding domains from collagen, laminin, etc.^87^, growth factor peptides from bone, platelets, etc.^88,89^, crosslinking systems like SpyTag-SpyCatcher, nitric oxide-cleavable crosslinker, etc.^90,91^, can potentially lead us a step closer to clinical and therapeutic realization. Moreover, systematic study of programming molecular self-assembly in modular RLP-based systems by extending the design rules applied for IDPs, such as combining two peptide properties into one sequence by increasing fraction of doping and varying block length, and/or blending at varying volume fractions can provide a new platform for design and application of RLP-based materials for intracellular material manipulation^34,92^. In addition, introduction of non-canonical amino acids, such as 4S-fluoroproline and thiazolidine carboxylic acid (isostructural analogues of proline)^93^, and/or post-translational modifications, such as conversion of proline to hydroxyproline, and tyrosine to DOPA^94^ could also be potentially applied to expand this design space (increase in functional diversity and development of new RLP-based hybrid biomaterials) with useful properties for applications in nanobiotechnology and medicine.

On the other hand, biofabrication of modular RLP-based biomaterials for in vivo application is still in its infancy, where the use of advanced fabrication strategies^95^, including electrospinning, extrusion-based 3D printing and soft lithography, can potentially expand the application of RLPs and modular RLP-based hydrogels in tissue engineering and regenerative medicine. A versatile protein-engineered ink platform with multiphase, multi-material and multiscale advanced manufacturing methods: specifically, the multi-stimuli-responsive behaviour of RLPs has tremendous potential for development of 4D printed shape-memory hydrogels for a variety of applications including microfluidics and soft-robotics^96^. Moreover, the current flow property limitations (low viscosity and non-shear thinning behaviour) of pristine resilin-based inks for 3D/4D printing can be addressed through strategies, such as modular protein engineering with amphiphilic tetrablock recombinamer and leucine zipper domains^97^, cysteine residue incorporation in peptide backbone, and/or blending with shear thinning protein polymer solutions^98^. Recently, soft and elastic hydrogel actuators made of synthetic polymers, which reversibly change their shape or dimension when exposed to external stimuli (pH, temperature, ions, light, electric and magnetic fields)^99^, have gained increasing research attention in implantable neural interfaces and neuromodulation owing to their shock absorption and vibration dampening properties^100^. Such systems have the ability to reduce micromotion-induced damage or strain to tissues, and support in vitro neuron attachment and growth better than traditional stiff microelectrodes; however, they suffer from poor biocompetency^101^. Biomimetic protein-based soft elastomeric hydrogels, which exhibit excellent biocompatibility and mechanical properties close to that of biological tissues, can serve as potential alternatives to synthetic polymers^101,102^. On this basis, RLPs exhibiting several specific advantages over other biomimetic polypeptides, including multi-stimuli responsiveness, soft and outstanding mechanical resilience, are perfect candidates for such specific and delicate applications. Design and development of new RLP-based elastic and self-healing hydrogels could potentially expand their applications in the field of neural tissue engineering, drug delivery, hydrogel bioelectronics and regenerative medicine with tuneable topological, biological and mechanical properties. A bright future exists for RLP-based materials for soft neurotechnology for efficient and effective communication between medical devices and human tissue for treatment of neurological disorders. In the field of neural tissue engineering, there is an unmet clinical need to address the complex biological process of nerve regeneration and develop effective medical treatment for central nervous system repair^103^. As an innovative material solution, RLPs hold significant potential for minimizing the physical and mechanical mismatch between neural tissues and implantable interfaces. Fabrication of soft implants with brain-like materials is not yet possible^104^; however, if RLP’s flexibility, elasticity, can be coupled with advanced fabrication or miniaturization to cellular or sub-cellular dimensions, along with different types of transducers, then it could be possible to engineer and develop soft neural implants with unprecedented biological modalities. Moreover, the remarkable sets of rare properties and unparallel functionality of RLPs make them unique for advanced drug delivery and therapeutic applications. The advantages of fusing therapeutic peptide to RLP-based carriers have the potential for a local therapy with improved delivery efficiency due to their temperature sensitivity and ability to self-assemble. Recently, Liu and co-workers^70^ demonstrated the potential of using these smart hydrogels in a variety of applications ranging from scaffolds for tissue engineering to drug-delivery systems that target the intracellular reductive environments of tumours using redox-responsive hydrogels. RLP gels could be also tailored as nitric oxide (NO)-scavenging gel with NO-cleavable crosslinker, which has potential as a therapeutic material for various chronic inflammatory disorders (e.g, rheumatoid arthritis) when NO levels are upregulated for a long time^91^. In the field of bio-adhesives, there is an unmet clinical need for biomimetic surgical glues that can provide flexibility without compromising strength and can stop body fluid and air leakages in different procedures such as lung and cardiovascular surgeries. RLP-based materials hold significant potential for development of strong sticky hydrogel platform that mimics the adhesion and biomechanical properties of a variety of native tissue microenvironments.

**Fig. 5:**
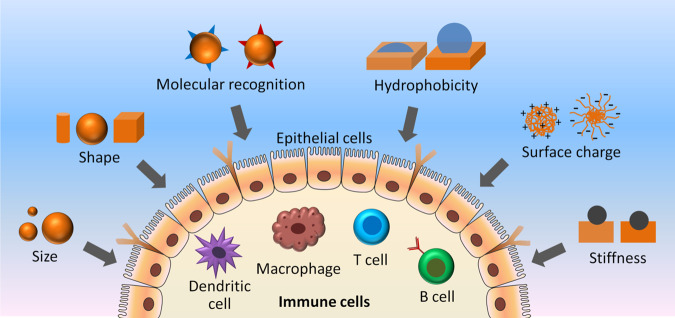
Schematic of design consideration for RLP-based smart nanoparticle and hydrogel systems to enable immunomodulation. Design elements, including size, shape, molecular recognition, hydrophobicity, surface charge and stiffness of RLP-based biomaterials can modulate their immune cell (dendritic cells, macrophages, T cells, B cells, etc.) interaction and molecular response.

Over the last decade, nanoengineered hydrogels and scaffolds have found potential applications as immunomodulatory antigenic agents, which induce regulatory adjustment of the immune system to treat a broad spectrum of diseases^105^. In this context, RLP-based biomaterials (macromers, NPs and gels) have the potential to tackle the biological delivery challenges and weak immunogenicity of many antigens in order to target antigen-presenting cells (APCs) in new vaccination strategies. Due to their biodegradability, biocompatibility and lack of immunogenicity, RLP-based biomaterials can be excellent carriers in vaccine delivery and, with novel chemical strategies, can be employed to release antigen intracellularly, enabling progress toward rational vaccine design. In this context, several design parameters, including size, shape, hydrophobicity, stiffness, surface charge and molecular recognition (target specificity), can be potentially applied to modular RLP-based biomaterials in the future to examine their potential in regulating the kinetics of multiple steps in the immune response (Fig. 5), including formation of fibrotic capsule, differential activation of dendritic cells, interplay with adaptive immune cells, recognition and removal by antibody and complement proteins^106^. By controlling the size and shape of modular RLP-based hydrogels and/or NPs, one can possibly regulate their in vivo permeability, distribution, transport and retention (which are subjected to translational and rotational motion), where NPs >200 nm often undergo heterogeneous distribution, whereas between 20 and 50 nm passively drain through lymphatics^107,108^. Moreover, the endocytosis pathway and APC activation can also be possibly modulated by controlling the size and shape of modular RLP-based hydrogels and/or NPs, where, in general, spherical shape results as largely membrane bound, and considered favourable over nanorods and nanochains^107,108^. Conversely, by tuning the surface roughness and hydrophobicity of modular RLP-based hydrogels and/or NPs, one can possibly control the impact of immune recognition, activity and interplay, where scaffolds with rough surface often show increased inflammasome activity compared to smooth surface, and scaffolds with hydrophobic surface often show increased gene expression of pro-inflammatory cytokines^109,110^. In addition, by tuning the surface charge and stiffness of modular RLP-based hydrogels and/or NPs, one can also possibly control their distribution/accumulation and endocytosis, where positively charged NPs tend to accumulate in the liver hepatocytes and negatively charged particles often show nonspecific distribution in the liver^111^. On the other hand, soft hydrogels are energetically less prone to full wrapping than stiff ones^112^. Furthermore, receptor-mediated endocytosis and antibody labelling of modular RLP-based hydrogels and/or NPs can also provide efficient in vivo imaging of target cells^113^. Therefore, future investigations on immune response control using modular RLP-based hydrogels and/or NPs (with a wide range of design control parameters applied) as antigenic agents in the presence and absence of other immunomodulatory factors can offer advancements in the field of RLP-research. Finally, such endeavour will offer a new platform for design and development of novel smart biomimetic protein-based materials for a wide range of biomedical applications, making their clinical and therapeutic applications a reality in near future. Conversely, the recent success in expression of RLPs in transgenic plants to improve the elastic modulus and toughness of stems has opened a new platform for application of RLPs in the plant kingdom^114^.

The unique amino acid consensus sequence, multi-stimuli responsiveness and molecular flexibility of RLPs have demonstrated potential to facilitate the programmed synthesis, akin to bio-mineralization of inorganic nanostructures, targeting both monometallic and alloy NPs of controlled composition, size, shape and morphology^53^. Even within the areas of alloy design the technique can support synthesis of ‘high-entropy alloys’ (multi-component alloys, supported with more than five elements and the designs are based on near equiatomic molar ratio), which belong to a promising category with characteristics like cocktail effects with severe lattice distortion^115^. Indeed, the multi-dimensional compositional space that can be tackled with this approach is practically limitless. For example, in such bio-nanoconjugates the surface-bound RLP-ligands can also be designed to incorporate receptor binding and/or therapeutic components to further expand the activity, to design and develop novel NPs consisting of both diagnostic and therapeutic components, which has a potential capability of both delivering therapy and self-reporting/tracking disease through bioimaging (using fluorescent sub-nano noble metal nanoclusters)^116^. Novel NPs with advanced magnetic properties can also be pursued to make better MRI probes and to visualize biological events. In fact, it has potential to deliver several different types of imaging agents to perform multimodality imaging. Optically coupled FMNC–RLP nanobioconjugates have the unique potential to display dual fluorescence emissions—one from the FMNCs and the other from RLPs, which can be potentially applied as metal-ion modulated ratiometric fluorescence probe array for the detection and identification of amino acids^117^. The intensities of the two fluorescence peaks can be modulated differently via specific environment and metal ions interaction with RLPs, which offer four different possible fluorescence responses, such as double enhancement/quenching and one enhancement/quenching the other^117^. Moreover, the fluorescence emission of optically coupled FMNC–RLP nanobioconjugates can also be tuned to near-infrared wavelength (700–900 nm) by controlling the size of FMNCs being synthesized^53^, and potentially applied as novel contrast imaging agents for fluorescence imaging of tumours, where non-covalently stabilized ultrasmall FMNCs can be largely accumulated in the tumour sites due to enhanced permeability and retention effects^118^. The unique and controllable fluorescence properties of FMNCs in combination with state-of-the-art intraoperative image systems have the potential to benefit image-guided surgical outcomes providing detection up to several millimetres deep into tissue and reducing positive margins^118^. Such bioinspired methods, using highly flexible and multi-stimuli-responsive RLPs as the driver of the bio-mineralization process, offer a powerful opportunity for sustainable and environmentally friendly production of complex inorganic nanomaterials, potentially on large scales, where the properties can be directly tuned by both the synthesis conditions and the RLP molecules used to generate controlled inorganic nano-/sub-nanostructures^53^. Moreover, atomic-level control on the fabrication of inorganic nanostructures and nanobioconjugates cannot be readily achievable using conventional approaches, and could potentially be adapted for diverse array of applications including optics/plasmonics, catalysis, novel biomarkers for inflammatory diseases, biological and chemical sensors, energy conversion/harvesting and storage, therapeutic delivery, diagnostics and theragnostics. However, fundamental research questions still remain (Box 2) that needs to be addressed for a better understanding and future development of novel RLP-based biomaterials.

Box 2 Future research questions

- What is the minimum glycine and proline residue content required for structural disorder and stimuli responsiveness in RLPs?
- What is the minimum length of amino acid sequence required for multi-stimuli responsiveness in RLPs?
- How dual-phase transition behaviour is encoded in the structure, and in the amino acid sequence level?
- Can expressing RLPs in animal cells provide advancements and advantages in the field?
- How does the physicochemical properties of secondary domains influence structure–property relationship of modular RLP-based biomaterials?
- How does different design parameters, such as size, shape, hydrophobicity, molecular recognition, surface charge and stiffness of of RLP-based biomaterials influence their immunogenecity?

## References

[1] Chen Q, Liang S, Thouas GA (2013). Elastomeric biomaterials for tissue engineering. Prog. Polym. Sci..

[2] Weis-Fogh T (1960). A rubber-like protein in insect cuticle. J. Exp. Biol..

[3] Bailey K, Weis-Fogh T (1961). Amino acid composition of a new rubber-like protein, resilin. Biochim. Biophys. Acta.

[4] Andersen SO (1964). The crosslinks in resilin identified as dityrosine and trityrosine. Biochim. Biophys. Acta.

[5] Elliott GF, Huxley AF, Weis-Fogh T (1965). On the structure of resilin. J. Mol. Biol..

[6] Coles GC (1966). Studies on resilin biosynthesis. J. Insect Physiol..

[7] Kannupandi T (1976). Occurrence of resilin and its significance in the cuticle of Pennella elegans, a copepod parasite. Acta Histochem..

[8] Edwards HA (1983). Occurrence of resilin in elastic structures in the food-pump of reduviid bugs. J. Exp. Biol..

[9] Skals N, Surlykke A (1999). Sound production by abdominal tymbal organs in two moth species: the green silver-line and the scarce silver-line (Noctuoidea: Nolidae: Chloephorinae). J. Exp. Biol..

[10] Michels J, Appel E, Gorb SN (2016). Functional diversity of resilin in Arthropoda. Beilstein J. Nanotechnol..

[11] Ardell DH, Andersen SO (2001). Tentative identification of a resilin gene in Drosophila melanogaster. Insect Biochem. Mol. Biol..

[12] Elvin CM (2005). Synthesis and properties of crosslinked recombinant pro-resilin. Nature.

[13] Kim M, Elvin C, Brownlee A, Lyons R (2007). High yield expression of recombinant pro-resilin: lactose-induced fermentation in *E. coli* and facile purification. Protein Eng. Des. Sel..

[14] Su RSC, Kim Y, Liu JC (2014). Resilin: protein-based elastomeric biomaterials. Acta Biomater..

[15] Balu R, Whittaker J, Dutta NK, Elvin CM, Choudhury NR (2014). Multi-responsive biomaterials and nanobioconjugates from resilin-like protein polymers. J. Mater. Chem. B.

[16] Vashi AV (2013). Controlled surface modification of tissue culture polystyrene for selective cell binding using resilin-inspired polypeptides. Biofabrication.

[17] Charati MB, Ifkovits JL, Burdick JA, Linhardt JG, Kiick KL (2009). Hydrophilic elastomeric biomaterials based on resilin-like polypeptides. Soft Matter.

[18] DiMarco RL, Heilshorn SC (2012). Multifunctional materials through modular protein engineering. Adv. Mater..

[19] Lau HK, Kiick KL (2015). Opportunities for multicomponent hybrid hydrogels in biomedical applications. Biomacromolecules.

[20] Wang Y, Katyal P, Montclare JK (2019). Protein-engineered functional materials. Adv. Healthc. Mater..

[21] Uversky VN, Gillespie JR, Fink AL (2000). Why are “natively unfolded” proteins unstructured under physiologic conditions?. Proteins.

[22] Lyons RE (2011). Molecular and functional characterisation of resilin across three insect orders. Insect Biochem. Mol. Biol..

[23] Lyons RE (2009). Comparisons of recombinant resilin-like proteins: repetitive domains are sufficient to confer resilin-like properties. Biomacromolecules.

[24] Tamburro AM (2010). Molecular and supramolecular structural studies on significant repetitive sequences of resilin. ChemBioChem.

[25] Andersen SO (2010). Studies on resilin-like gene products in insects. Insect Biochem. Mol. Biol..

[26] Oates ME (2013). D^2^P^2^: database of disordered protein predictions. Nucleic Acids Res..

[27] Szklarczyk D (2018). STRING v11: protein–protein association networks with increased coverage, supporting functional discovery in genome-wide experimental datasets. Nucleic Acids Res..

[28] van der Lee R (2014). Classification of intrinsically disordered regions and proteins. Chem. Rev..

[29] Balu R (2014). An16: an advanced multi-stimuli-responsive resilin-mimetic protein polymer. Acta Biomater..

[30] Balu R (2015). Structural ensembles reveal intrinsic disorder for the multi-stimuli responsive bio-mimetic protein Rec1. Sci. Rep..

[31] Balu R (2016). Effects of crowding and environment on the evolution of conformational ensembles of the multi-stimuli-responsive intrinsically disordered protein, Rec1: a small-angle scattering investigation. J. Phys. Chem. B.

[32] Quiroz FG, Chilkoti A (2015). Sequence heuristics to encode phase behaviour in intrinsically disordered protein polymers. Nat. Mater..

[33] Löwik DWPM, Leunissen EHP, van den Heuvel M, Hansen MB, van Hest JCM (2010). Stimulus responsive peptide based materials. Chem. Soc. Rev..

[34] Dzuricky M, Rogers BA, Shahid A, Cremer PS, Chilkoti A (2020). De novo engineering of intracellular condensates using artificial disordered proteins. Nat. Chem..

[35] Truong MY (2010). A pH-responsive interface derived from resilin-mimetic protein Rec1. Biomaterials.

[36] Dutta NK (2011). A Genetically engineered protein responsive to multiple stimuli. Angew. Chem. Int. Ed..

[37] Mayavan S (2011). Self-organization, interfacial interaction and photophysical properties of gold nanoparticle complexes derived from resilin-mimetic fluorescent protein Rec1. Biomaterials.

[38] Meyer DE, Chilkoti A (2004). Quantification of the effects of chain length and concentration on the thermal behavior of elastin-like polypeptides. Biomacromolecules.

[39] Li L, Luo T, Kiick KL (2015). Temperature-triggered phase separation of a hydrophilic resilin-like polypeptide. Macromol. Rapid Commun..

[40] Whittaker JL (2015). Tunable thermoresponsiveness of resilin via coassembly with rigid biopolymers. Langmuir.

[41] Bracalello A (2011). Design and production of a chimeric resilin-, elastin-, and collagen-like engineered polypeptide. Biomacromolecules.

[42] Lyons RE, Elvin CM, Taylor K, Lekieffre N, Ramshaw JAM (2012). Purification of recombinant protein by cold-coacervation of fusion constructs incorporating resilin-inspired polypeptides. Biotechnol. Bioeng..

[43] Miyawaki A, Niino Y (2015). Molecular spies for bioimaging—fluorescent protein-based probes. Mol. Cell.

[44] Renner JN, Cherry KM, Su RSC, Liu JC (2012). Characterization of resilin-based materials for tissue engineering applications. Biomacromolecules.

[45] Huang S-C (2017). Rational design and hierarchical assembly of a genetically engineered resilin–silk copolymer results in stiff hydrogels. ACS Biomater. Sci. Eng..

[46] Luo F, Qian Z-G, Xia X-X (2018). Responsive protein hydrogels assembled from spider silk carboxyl-terminal domain and resilin copolymers. Polymers.

[47] Xia X-X, Xu Q, Hu X, Qin G, Kaplan DL (2011). Tunable self-sssembly of genetically engineered silk–elastin-like protein polymers. Biomacromolecules.

[48] Weitzhandler I (2017). Micellar self-assembly of recombinant resilin-/elastin-like block copolypeptides. Biomacromolecules.

[49] Fang W (2018). Coacervation of resilin fusion proteins containing terminal functionalities. Colloids Surf. B.

[50] Dutta NK (2009). Physical approaches for fabrication of organized nanostructure of resilin-mimetic elastic protein rec1-resilin. Biomaterials.

[51] Dutta, N. K. et al. Template directed formation of metal nanoparticles and uses thereof. Patent WO2014071463A1 (2014).

[52] Balu R (2019). Evolution of the interfacial structure of a catalyst ink with the quality of the dispersing solvent: a contrast variation small-angle and ultra-small-angle neutron scattering investigation. ACS Appl. Mater. Interfaces.

[53] Dutta, N. K. et al. Formation of sub-nano metal particles. Patent WO2015024063A1 (2015).

[54] Balu R (2015). A multi-responsive intrinsically disordered protein (IDP)-directed green synthesis of fluorescent gold nanoclusters. J. Mater. Chem. B.

[55] Balu R (2019). A sustainable biomineralization approach for the synthesis of highly fluorescent ultra-small Pt nanoclusters. Biosensors.

[56] Griffo A (2017). Single-molecule force spectroscopy study on modular resilin fusion protein. ACS Omega.

[57] Rauscher S (2006). Proline and glycine control protein self-organization into elastomeric or amyloid fibrils. Structure.

[58] Kappiyoor R, Balasubramanian G, Dudek DM, Puri IK (2011). Elastomechanical properties of resilin. Soft Matter.

[59] Truong MY (2011). The effect of hydration on molecular chain mobility and the viscoelastic behavior of resilin-mimetic protein-based hydrogels. Biomaterials.

[60] Qin G (2011). Recombinant exon-encoded resilins for elastomeric biomaterials. Biomaterials.

[61] Qin G, Hu X, Cebe P, Kaplan DL (2012). Mechanism of resilin elasticity. Nat. Commun..

[62] Degtyar E, Mlynarczyk B, Fratzl P, Harrington MJ (2015). Recombinant engineering of reversible crosslinks into a resilient biopolymer. Polymer.

[63] Li L, Tong Z, Jia X, Kiick KL (2013). Resilin-like polypeptide hydrogels engineered for versatile biological function. Soft Matter.

[64] Li L, Teller S, Clifton RJ, Jia X, Kiick KL (2011). Tunable mechanical stability and deformation response of a resilin-based elastomer. Biomacromolecules.

[65] Li L (2016). Recombinant resilin-based bioelastomers for regenerative medicine applications. Adv. Healthc. Mater..

[66] Lv S (2010). Designed biomaterials to mimic the mechanical properties of muscles. Nature.

[67] Kim Y, Renner JN, Liu JC (2014). Incorporating the BMP-2 peptide in genetically-engineered biomaterials accelerates osteogenic differentiation. Biomater. Sci..

[68] Kim Y, Liu JC (2016). Protein-engineered microenvironments can promote endothelial differentiation of human mesenchymal stem cells in the absence of exogenous growth factors. Biomater. Sci..

[69] Kim Y, Gill EE, Liu JC (2016). Enzymatic crosslinking of resilin-based proteins for vascular tissue engineering applications. Biomacromolecules.

[70] Su RS-C, Galas RJ, Lin C-Y, Liu JC (2019). Redox-responsive resilin-like hydrogels for tissue engineering and drug delivery applications. Macromol. Biosci..

[71] McGann CL, Levenson EA, Kiick KL (2013). Resilin-based hybrid hydrogels for cardiovascular tissue engineering. Macromol. Chem. Phys..

[72] McGann CL, Akins RE, Kiick KL (2016). Resilin-PEG hybrid hydrogels yield degradable elastomeric scaffolds with heterogeneous microstructure. Biomacromolecules.

[73] Sanami M (2015). Biophysical and biological characterisation of collagen/resilin-like protein composite fibres. Biomed. Mater..

[74] Whittaker JL, Dutta NK, Elvin CM, Choudhury NR (2015). Fabrication of highly elastic resilin/silk fibroin based hydrogel by rapid photo-crosslinking reaction. J. Mater. Chem. B.

[75] McGann CL, Dumm RE, Jurusik AK, Sidhu I, Kiick KL (2016). Thiol-ene photocrosslinking of cytocompatible resilin-like polypeptide-PEG hydrogels. Macromol. Biosci..

[76] Lau HK, Li L, Jurusik AK, Sabanayagam CR, Kiick KL (2017). Aqueous liquid–liquid phase separation of resilin-like polypeptide/polyethylene glycol solutions for the formation of microstructured hydrogels. ACS Biomater. Sci. Eng..

[77] Lau HK (2018). Microstructured elastomer-PEG hydrogels via kinetic capture of aqueous liquid–liquid phase separation. Adv. Sci..

[78] Okesola BO (2020). Covalent co-assembly between resilin-like polypeptide and peptide amphiphile into hydrogels with controlled nanostructure and improved mechanical properties. Biomater. Sci..

[79] King RE (2019). Biocompatibility and viscoelastic properties of injectable resilin-like polypeptide and hyaluronan hybrid hydrogels in rabbit vocal folds. Regen. Eng. Transl. Med..

[80] Fang W (2017). Elastic and pH-responsive hybrid interfaces created with engineered resilin and nanocellulose. Biomacromolecules.

[81] Hu X, Xia X-X, Huang S-C, Qian Z-G (2019). Development of adhesive and conductive resilin-based hydrogels for wearable sensors. Biomacromolecules.

[82] Kragelund, B. B. & Skriver, K. *Intrinsically Disordered Proteins: Methods and Protocols* (Springer US, 2020). 10.1007/978-1-0716-0524-0_47 35178671

[83] Xue B, Mizianty M, Kurgan L, Uversky VN (2012). Protein intrinsic disorder as a flexible armor and a weapon of HIV-1. Cell. Mol. Life Sci..

[84] Goh GK-M, Keith DA, Foster JA, Uversky VN (2020). Rigidity of the outer shell predicted by a protein intrinsic disorder model sheds light on the COVID-19 (Wuhan-2019-nCoV) infectivity. Biomolecules.

[85] Su RSC, Renner JN, Liu JC (2013). Synthesis and characterization of recombinant abductin-based proteins. Biomacromolecules.

[86] Wei W (2016). An underwater surface-drying peptide inspired by a mussel adhesive protein. Adv. Funct. Mater..

[87] Rahmany MB, Van Dyke M (2013). Biomimetic approaches to modulate cellular adhesion in biomaterials: a review. Acta Biomater..

[88] Pierce GF, Mustoe TA, Altrock BW, Deuel TF, Thomason A (1991). Role of platelet-derived growth factor in wound healing. J. Cell. Biochem..

[89] Tao H (2015). BMP7-based functionalized self-assembling peptides for nucleus pulposus tissue engineering. ACS Appl. Mater. Interfaces.

[90] Zakeri B (2012). Peptide tag forming a rapid covalent bond to a protein, through engineering a bacterial adhesin. Proc. Natl Acad. Sci. USA.

[91] Yeo J (2019). Nitric oxide-scavenging nanogel for treating rheumatoid arthritis. Nano Lett..

[92] Simon JR, Carroll NJ, Rubinstein M, Chilkoti A, López GP (2017). Programming molecular self-assembly of intrinsically disordered proteins containing sequences of low complexity. Nat. Chem..

[93] Catherine C (2015). Engineering thermal properties of elastin-like polypeptides by incorporation of unnatural amino acids in a cell-free protein synthesis system. Biotechnol. Bioprocess Eng..

[94] Roberts S, Dzuricky M, Chilkoti A (2015). Elastin-like polypeptides as models of intrinsically disordered proteins. FEBS Lett..

[95] Pedde RD (2017). Emerging biofabrication strategies for engineering complex tissue constructs. Adv. Mater..

[96] Mitchell A, Lafont U, Hołyńska M, Semprimoschnig C (2018). Additive manufacturing—a review of 4D printing and future applications. Addit. Manuf..

[97] Salinas-Fernández, S., Santos, M., Alonso, M., Quintanilla, L. & Rodríguez-Cabello, J. C. Genetically engineered elastin-like recombinamers with sequence-based molecular stabilization as advanced bioinks for 3D bioprinting. *Appl. Mater. Today* **18**, 100500 (2019).

[98] Costa JB (2019). Engineering patient-specific bioprinted constructs for treatment of degenerated intervertebral disc. Mater. Today Commun..

[99] Shi Q (2019). Bioactuators based on stimulus-responsive hydrogels and their emerging biomedical applications. NPG Asia Mater..

[100] Feiner R, Dvir T (2017). Tissue–electronics interfaces: from implantable devices to engineered tissues. Nat. Rev. Mater..

[101] Wang E, Desai MS, Lee S-W (2013). Light-controlled graphene-elastin composite hydrogel actuators. Nano Lett..

[102] Zhang Y, Desai MS, Wang T, Lee S-W (2020). Elastin-based thermoresponsive shape-memory hydrogels. Biomacromolecules.

[103] Song S, George PM (2017). Conductive polymer scaffolds to improve neural recovery. Neural Regen. Res..

[104] Lacour SP, Courtine G, Guck J (2016). Materials and technologies for soft implantable neuroprostheses. Nat. Rev. Mater..

[105] Singh A, Peppas NA (2014). Hydrogels and scaffolds for immunomodulation. Adv. Mater..

[106] Andorko JI, Jewell CM (2017). Designing biomaterials with immunomodulatory properties for tissue engineering and regenerative medicine. Bioeng. Transl. Med..

[107] Kobayashi H, Watanabe R, Choyke PL (2014). Improving conventional enhanced permeability and retention (EPR) effects; what is the appropriate target?. Theranostics.

[108] Huang X (2011). The shape effect of mesoporous silica nanoparticles on biodistribution, clearance, and biocompatibility in vivo. ACS Nano.

[109] Vaine CA (2013). Tuning innate immune activation by surface texturing of polymer microparticles: the role of shape in inflammasome activation. J. Immunol..

[110] Moyano DF (2012). Nanoparticle hydrophobicity dictates immune response. J. Am. Chem. Soc..

[111] Elci SG (2016). Surface charge controls the suborgan biodistributions of gold nanoparticles. ACS Nano.

[112] Anselmo AC (2015). Elasticity of nanoparticles influences their blood circulation, phagocytosis, endocytosis, and targeting. ACS Nano.

[113] Burrows M, Borycz JA, Shaw SR, Elvin CM, Meinertzhagen IA (2011). Antibody labelling of resilin in energy stores for jumping in plant sucking insects. PLos ONE.

[114] Preis I, Abramson M, Shoseyov O (2018). The modification of cell wall properties by expression of recombinant resilin in transgenic plants. Mol. Biotechnol..

[115] George EP, Raabe D, Ritchie RO (2019). High-entropy alloys. Nat. Rev. Mater..

[116] Hu Y, Guo W, Wei H (2015). Protein- and peptide-directed approaches to fluorescent metal nanoclusters. Isr. J. Chem..

[117] Dutta, N. K., Dutta, A. K. & Choudhury, N. R. in *Encyclopedia of Metalloproteins* (eds. Kretsinger, R. H., et al.) (Springer, New York, 2013).

[118] Hill TK, Mohs AM (2016). Image-guided tumor surgery: will there be a role for fluorescent nanoparticles?. Wiley Interdiscip. Rev. Nanomed. Nanobiotechnol..

